# Biocompatibility of Nanoscale Hydroxyapatite Coating on TiO_2_ Nanotubes

**DOI:** 10.3390/ma12121979

**Published:** 2019-06-20

**Authors:** Xiaokai Zhang, Dechuang Zhang, Qing Peng, Jianguo Lin, Cuie Wen

**Affiliations:** 1 School of Materials Science and Engineering, Xiangtan University, Xiangtan 411105, China; zhangxiaokaixtu@163.com; 2 Key Laboratory of Materials Design and Preparation Technology of Hunan Province, Xiangtan University, Xiangtan 411105, China; pengqingek@163.com; 3 School of Aerospace, Mechanical and Manufacturing Engineering, RMIT University, Melbourne, Victoria 3083, Australia; cuie.wen@rmit.edu.au

**Keywords:** TiO_2_ nanotubes, HA coating, bond strength, biocompatibility, mesenchymal stem cells

## Abstract

In this study, a highly-ordered TiO_2_ nanotube array was successfully fabricated on the surface of a pure titanium foil using the anodization method, and a hydroxyapatite (HA) layer was electrochemically deposited on the vertically aligned titania (TiO_2_) nanotube array. The TiO_2_ nanotubes exhibited an inner diameter ranging from 44.5 to 136.8 nm, a wall thickness of 9.8 to 20 nm and a length of 1.25 to 3.94 µm, depending on the applied potential, and the anodization time and temperature. The TiO_2_ nanotubes provided a high number of nucleation sites for the HA precipitation during electrochemical deposition, resulting in the formation of a nanoscale HA layer with a particle size of about 50 nm. The bond strength between the HA coating and the nanotubular layer with an inner diameter of 136.8 nm was over 28.7 MPa, and the interlocking between the nanoscale HA and the TiO_2_ nanotubes may have been responsible for the high bond strength. The biocompatibility assessment was conducted on Ti foil with a composite coat of nanoscale HA and the TiO_2_ nanotube array by 3-(4,5-dimethylthiazol-2-yl)-2,5-diphenyltetrazolium bromide (MTT) array with mesenchymal stem cells (MSCs). The mesenchymal stem cells adhered to and spread onto the nanoscale HA layer with plenty of extending filopodia, indicating excellent biocompatibility of the HA coat, the composite coat of nanoscale HA and the TiO_2_ nanotube array. The findings suggest that the nanoscale HA coating on the TiO_2_ nanotube array might be a promising way to improve the bond strength and the compatibility of the HA layer.

## 1. Introduction

Biomedical metal materials, such as precious metals, stainless steels, cobalt alloys, and titanium alloy materials, have been widely used in the field of hard tissue replacement due to their high strength and toughness, excellent fatigue resistance, and good processing performance [1]. Titanium and titanium alloys have better mechanical compatibility than other three alloys and are currently the most promising metal materials for clinical applications. It was found that titanium and titanium alloy implants are biologically inert materials that cannot be distinguished by the immune system in living organisms [2]. A layer of cystic fiber membrane is formed on the surface of the titanium implant to shield the stimulation, so titanium alloy implants are difficult to organically combine with the bone tissue [3]. If only short-term, metal implants are acceptable. In many cases, however, long-term implantation of metal implants is required. For example, fractures require long-term placement of the metal [4]. Therefore, it is necessary to increase the biocompatibility of the titanium alloy to achieve an organic combination of the implant and the bone tissue.

Hydroxyapatite (HA, chemical formula, Ca_10_(PO_3_)_6_(OH)_2_) is a biologically active substance [5]. It is found that hydroxyapatite can be perfectly combined with the human body as an implant [6]. This is because hydroxyapatite can induce the deposition of calcium and phosphorus ions at the surface to form chemical bonding with the host bone tissue. More importantly, hydroxyapatite can be artificially synthesized, greatly expanding its application in the biomedical field. As a result, hydroxyapatite has already become an attractive coating material to accelerate bone growth around the implant. Many methods have been developed to deposit an HA layer onto biomedical metal surfaces, including plasma spraying [7], sputtering process [8], sol-gel method [9], pulsed laser deposition [10], and electrophoretic deposition [11]. However, the coating spallation from the substrate results in adverse clinical responses to the implants and surrounding tissue due to poor adhesion strength between the metal substrate and coating layer [12]. Thus, surface modification is essential since hydroxyapatite coating cannot be added during manufacturing processes such as casting, melting, forging, and heat treatment.

Based on bionics, nanomaterials with similar structures and properties of physiological tissues prepared by surface modification can greatly improve the biocompatibility of materials [13]. In recent years, the preparation of TiO_2_ nanotube coating has been a research hotspot [14]. The unique hollow tubular arrangement of TiO_2_ nanotubes greatly increases the surface roughness, gives it a large specific surface area and high adsorption capacity, and better deposits biomaterial coatings with better biocompatibility [15]. At present, the methods for preparing TiO_2_ nanotubes on the surface of titanium and titanium alloy are: the template method [16], alkali heat treatment [17] and electrochemical anodization [18]. In particular, researchers have done a lot of work in the preparation of TiO_2_ nanotubes by anodization. Park [19] successfully prepared an ordered array of TiO_2_ nanotubes on the titanium surface by anodization and evaluated the biological activity of TiO_2_ nanotubes in simulated body fluids. As a surface modification method, the anodic oxidation method is easy to operate, low cost, and can adapt to various complicated metal surfaces [20]. At the same time, the prepared TiO_2_ nanotube oxide layer has strong controllability and high bonding strength. 

In this study, a highly-ordered TiO_2_ nanotube array was successfully fabricated on the surface of a pure titanium foil using the anodization method, and a hydroxyapatite (HA) layer was electrochemically deposited on the vertically aligned titania (TiO_2_) nanotube array. The effect of the inner diameters on the bond strength between the nanoscale HA and the TiO_2_ nanotubes was investigated. The biocompatibility of the nanostructured surface was assessed using mesenchymal stem cells. 

## 2. Materials and Methods

### 2.1. Preparation of TiO_2_ Nanotube Array

Titanium foils with 99.9% purity were used as starting materials. Prior to anodic oxidation, the samples were ultrasonically cleaned in ethanol and acetone for 20 min each and then dried in a nitrogen stream. After degreasing, the samples were exposed to a solution of HF–HNO_3_–H_2_O at a volume ratio of 1:1:2 to etch the surface for 30 s. Finally, the samples were cleaned in deionized water and dried in air. The nanotubes on the Ti foil samples were anodized using an electrolyte with glycerol and water (volume ratio 1:1) containing 0.3 mol/L ammonium fluoride (NH_4_F) at an applied potential ranging from 10 to 40 V in a water bath. After the anodization, the samples were immediately rinsed in deionized water and dried in air. The nanotubes were annealed at 550 °C for 3 h in air at a heating rate of 10 min^−1^ to obtain the crystalline phase of TiO_2_. 

### 2.2. Hydroxyapatite Coating

Electrochemical deposition of the HA coating on the TiO_2_ nanotubes was conducted at a constant current density of 3 mA/cm^2^ at 50 °C in a water bath. The electrolyte contained 0.084 mol/L Ca(NO_3_)_2_, 0.05 mol/L (NH_4_)_2_HPO_4_, 0.1 mol/L NaNO_3_ and 0.0024 mol/L Na_3_C_6_H_5_O_7_. The Ca:P ratio of the electrolyte was maintained at 1.67 and the pH value was adjusted to 4.5–5.0 using HNO_3_ and NH_3_·H_2_O. After deposition, the samples were washed in a 0.1 mol/L NaOH solution for 2 h at 80 °C using an external heating system and then rinsed with deionized water and dried in air. 

### 2.3. Biocompatibility Assessment

MTT assay was carried out on bare Ti, TiO_2_ nanotubes and the nanoscale HA coating on TiO_2_ nanotubes using mesenchymal stem cells (MSCs) (Cell Resource Center of Shanghai Institute of Life Science). Bare titanium specimens were used as a control. Industrial pure titanium sheets (≥99.5% purity) were cut into pieces by a wire electric discharge machine, ground with SiC sandpaper of 600#, 800#, and 1000#, and then mechanically polished. Under ultrasonic conditions, the titanium sheets were washed in deionized water, absolute alcohol, and acetone for 20 min to completely dissolve the residual grease on the surface. After the degreasing treatment, the titanium sheet was rinsed with deionized water and then air-dried. The surface of the titanium was pretreated for 30 s in a mixed solution of HF, HNO_3_ and water at a volume ratio of 1:1:2, and then deionized. The surface was rinsed with water and dried for use.

The cells were cultured in low glucose Dulbecco’s modified Eagle medium (DMEM) with 30% neonate bovine serum and 3.7 g/L sodium bicarbonate to adjust the acid/base balance (Sigma, AR, St. Louis, MO, USA). The medium was changed every 2 day and the cell culture was carried out in an incubator at 35 °C with 5% CO_2_ and saturated humidity. The mesenchymal stem cells were seeded on each disc at a density of 1 × 10^4^ and cultured for 3 and 7 days.

After 7 days of culture on the sample, the cells were fixed with 3% glutaraldehyde for 2 h, then washed three times with phosphate buffered saline (PBS), fixed with 1% acid for one hour, and washed three times with PBS for 10 minutes each. Then, all samples were dehydrated with a gradient of absolute ethanol solution at 30%, 50%, 70%, 90%, and 100% for 10 min, and finally placed in a freeze dryer to dry the water in the mesenchymal stem cells. The dehydrated and fixed cells were observed by scanning electron microscopy (SEM) after gold spraying. 

A Cell Counting KIT-8 (CCK-8, Dojindo Co., Kumamoto, Japan) was used to measure the cell viability. The cell viability of the MC3T3-E1 cells of the Ti/TiO_2_/nHA coating was calculated according to the following formula [21]:

Cell viability (%) = [(OD_t_ − OD_blank_)/(OD_nc_ − OD_blank_)] × 100%,

where OD_t_ is the absorbance value of the experimental group, OD_nc_ is the absorbance value of the negative control group, and OD_blank_ is the absorbance value of the α-MEM medium.

### 2.4. Characterization of Surface Properties

The phase structures present in the surface microstructure were identified by X-ray diffraction (XRD, Rigaku, Tokyo, Japan) using a Rigaku D/Max 2500PC diffractometer operated at 50 kV and 100 mA with Cu K radiation (=1.5406 nm) at room temperature. The morphology and size of the TiO_2_ nanotube array and HA coating were characterized using a scanning electron microscope (JSM-6480LV, JEOL, Tokyo, Japan) equipped with energy dispersive X-ray spectrometry (EDS) at 20 kV (XL30 S-FEG). The transmission electron microscopy (TEM, JEM-2100, JEOL, Tokyo, Japan) observations were conducted using a JEM-2100 operating at 160 kV. The samples for TEM observation were scraped off of the titanium layer with a knife. They were then dispersed in an ethanol solution, dropped on a TEM copper wire with a carbon film, put through vacuum-assisted drying, and placed in an electric sample stage. 

### 2.5. Characterization of Bond Strength of the Coating

**Figure 1:**
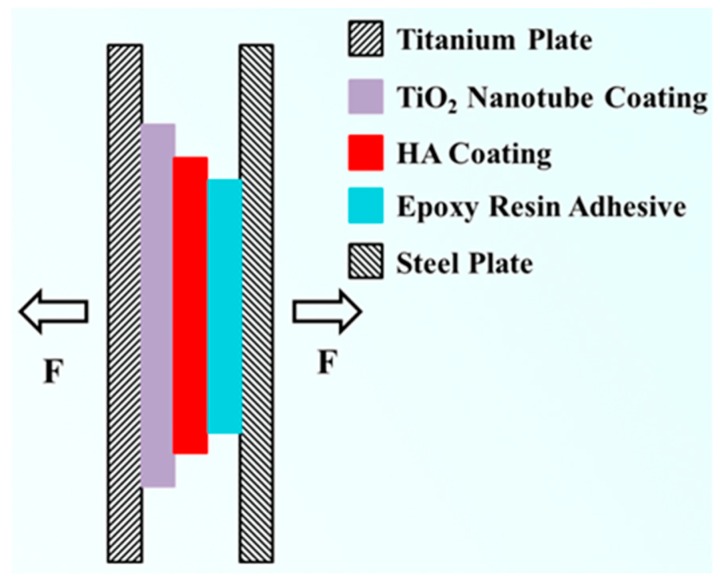
Stretching schematic diagram of Ti/TiO_2_/HA coating.

The bond strength of the coating was tested using an electronic universal testing machine (RG4100, Pioneer Times Technology Co., Ltd., Wuhan, China). The bond strength of the HA coating was measured via the modified ASTM C-633 method [22], and the test schematic is shown in Figure 1. The test procedure began with the selection of a thin steel sheet of 1 cm × 5 cm, which was polished. To reduce the surface chemical energy of the steel sheet, it was necessary to remove the oil by ultrasonic cleaning. Then, the thick coating of the steel plate was bonded with epoxy resin glue. The bonding area was 1 cm × 1 cm and the tensile test was performed after the epoxy resin had dried for 24 h. When the electronic universal testing machine was stretched, the loading speed was set to 1 mm/min, and the highest point of the curve, that is, the maximum pulling force, was recorded during the entire stretching process.

The combined strength calculation formula is as follows:

*σ*_b_ = *F*/*S*,

where *F* is the maximum tensile force, *S* is the bonding area, and *σ*_b_ is the bonding strength of the coating. 

The number of samples used for each test was three.

## 3. Results and Discussion

**Figure 2:**
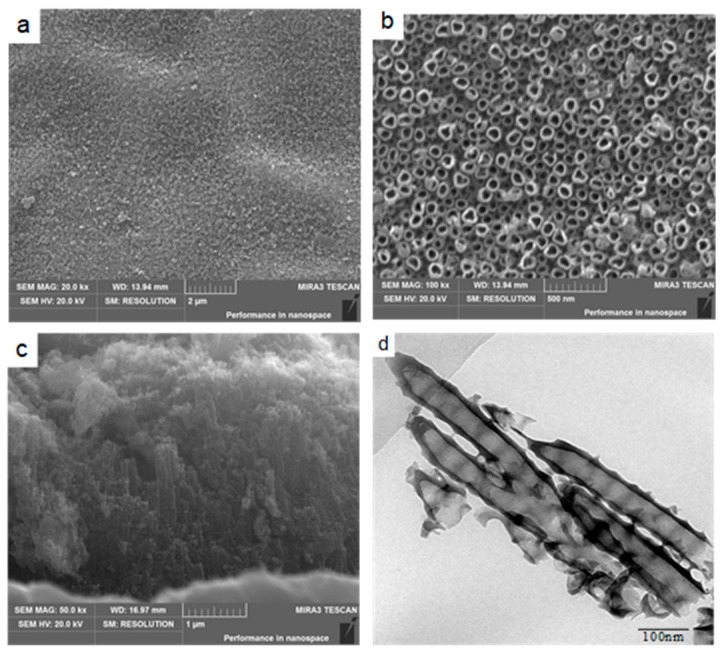
(**a**) SEM image of the TiO_2_ nanotube array formed by anodization in 0.3 mol/L NH_4_F + glycerol–water (volume ration 1:1) at 20 V, (**b**) higher magnifications of the TiO_2_ nanotube array, (**c**) cross-section of the TiO_2_ nanotube array, and (**d**) bright field TEM image of the nanotubes.

Figure 2a–c shows the SEM images of the top view and cross-section of TiO_2_ nanotubes anodized in 0.3 mol/L NH_4_F + glycerol–water (volume ratio 1:1) at 20 V Ti foil after 9 h anodization. The average nanotube diameter measured was 87.2 nm and the average thickness (or length of the nanotubes) was 3.4 μm. Close observation of the microstructures of the nanotubes were conducted using a TEM, and the bright field TEM image of the nanotubes is shown in Figure 2d. The nanotubes exhibited a hollow bamboo structure. This sort of hollow structure is beneficial for inducing the deposition of Ca^2+^ and PO_4_^3−^ ions, which will be discussed in a later section. In addition, the numerous nanotubes were expected to act as nucleation sites, thereby accelerating HA deposition and refining the HA grain crystals during electrochemical deposition [22].

**Figure 3:**
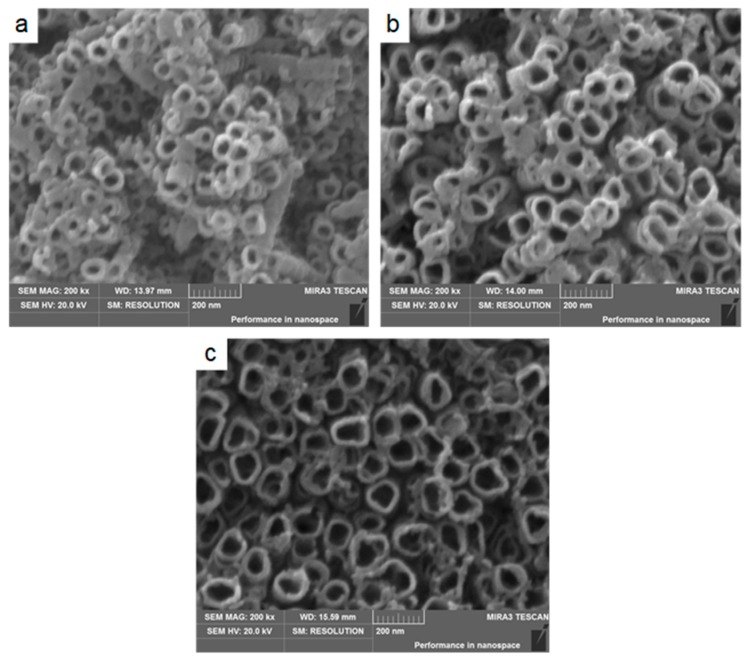
SEM images of nanotube arrays anodized at the following times: (**a**) 3 h, (**b**) 6 h and (**c**) 12 h, with inner diameters of approximately 44.5, 71.2 and 136.8 nm, respectively.

Moreover, to obtain TiO_2_ nanotubes with different dimensions (i.e., wall thickness and inner diameter), a different anodization time was used to prepare the nanotubes under the anodization voltage of 20 V. Figure 3 illustrates the top views of the morphologies of the nanotubes obtained at anodization times ranging from 3 to 12 h under the anodization voltage of 20 V. It was clear that prolonging the anodization time significantly increased the inner diameter but decreased the wall thickness. The nanotubes obtained by anodization at 20 V for 3 h had inner diameters of 44.5 nm and wall thicknesses of 20.0 nm (see Figure 3a), while the TiO_2_ nanotubes obtained by anodization at 20 V for 6 h had inner diameters of 71.2 nm and wall thicknesses of 14.4 nm (see Figure 3b). When the anodization time was prolonged to 12 h, the inner diameter of the TiO_2_ nanotubes further increased but the wall thickness further decreased, reaching 136.8 nm and 10.0 nm, respectively (see Figure 3c). The increasing tube diameter may have promoted the adsorption ability of the calcium and phosphorus ions of the highly ordered TiO_2_ arrays [23].

**Figure 4:**
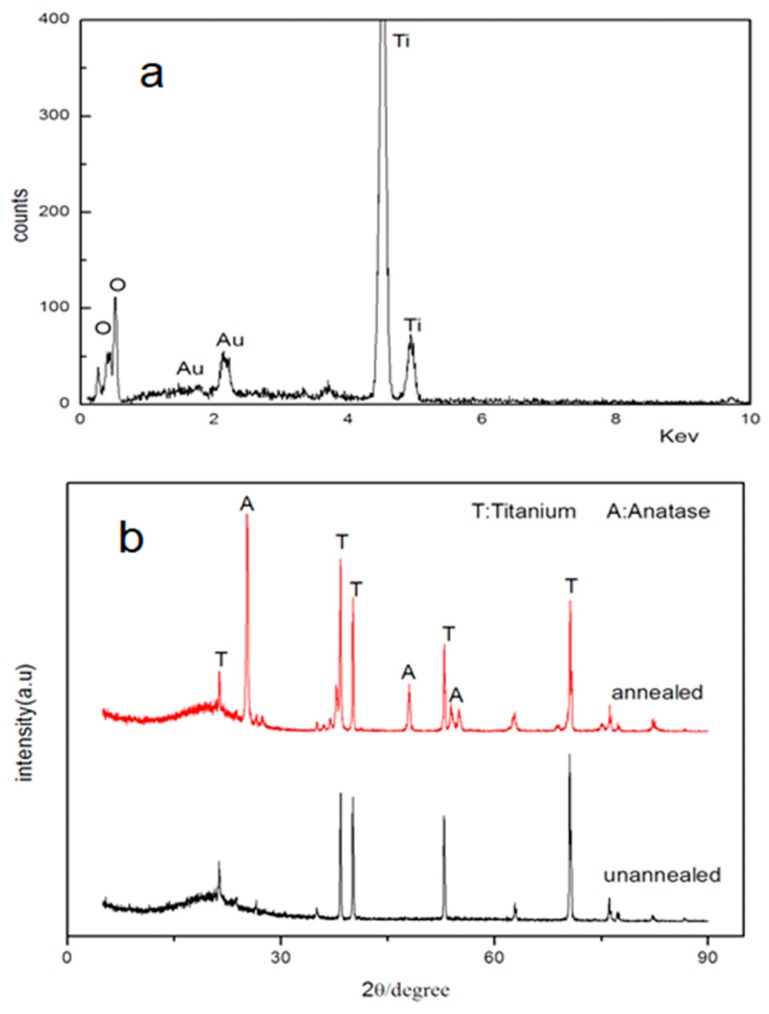
(**a**) EDS microanalysis of nanotubes and (**b**) XRD spectra of the nanotubular layers before and after annealing at 550 °C for 3 h.

Figure 4 presents the results of EDS microanalysis and X-ray diffraction measurements for the nanotubes after annealing at 550 °C for 3 h in air. The nanotubes were anodized with the use of NH_4_F + glycerol–water electrolyte at the applied potential of 20 V for 12 h. Only Ti and O peaks were detected in the nanotube array; and the O/Ti molar ratio calculated by EDS (Figure 4a) was 2.01, which indicates a stoichiometric TiO_2_. The XRD results in Figure 4b show that the as-formed nanotubular layer was an amorphous structure, which turned into an anatase structure after annealing in air at 550 °C for 3 h. 

**Figure 5:**
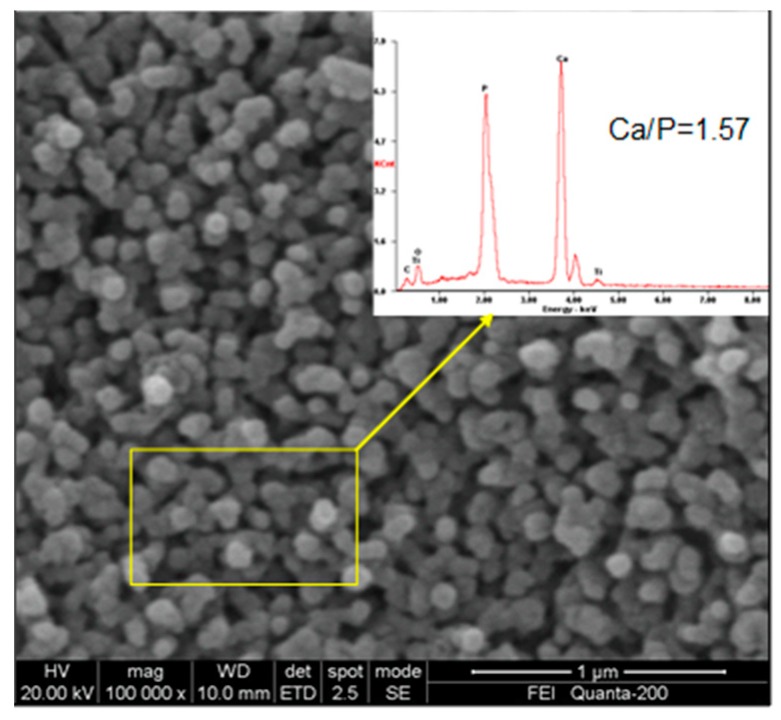
SEM image of an HA coating on TiO_2_ nanotubes; the insert refers to EDS microanalysis of the HA coating.

Figure 5 shows the SEM image of the HA coating electrochemically deposited on the TiO_2_ nanotubes at 3 mA/cm^2^ with the use of a calcium-phosphorus electrolyte and calcinated at 700 °C. The top view of the HA coating shows the HA particles with an average diameter of about 50 nm. The inserts refer to the EDS microanalysis of the HA coating, the value of Ca/P of the HA was calculated as 1.57, which was very close to the 1.67 standard stoichiometric HA. HA with a Ca/P ratio ~1.57 is reported to be more beneficial for bone formation [24].

**Figure 6:**
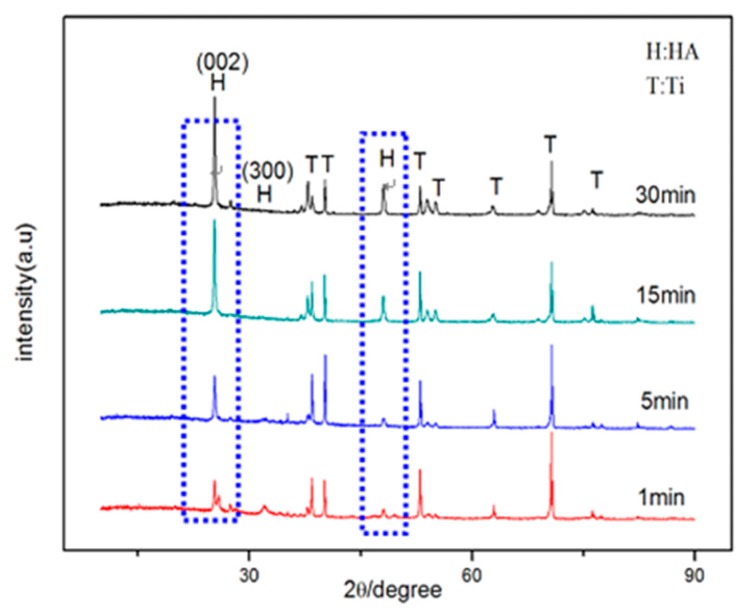
XRD spectra of HA coatings at various electrochemical deposition times from 1 min to 30 min.

Figure 6 presents the XRD patterns for the HA coatings on TiO_2_ nanotubes at different deposition times of 1 min, 5 min, 10 min, and 30 min. The peaks on the XRD patterns are well fitted to the standard spectrum of HA (ICDD No. 74-566). We noted that with the increase of deposition time, the content of the HA phase gradually increased, which was evidenced by the varying intensities of the peaks from the HA phases in the XRD profiles. However, we could not determine whether the growth of HA was in monoaxial mode according to the XRD pattern [25], so SEM characterization is carried out below.

**Figure 7:**
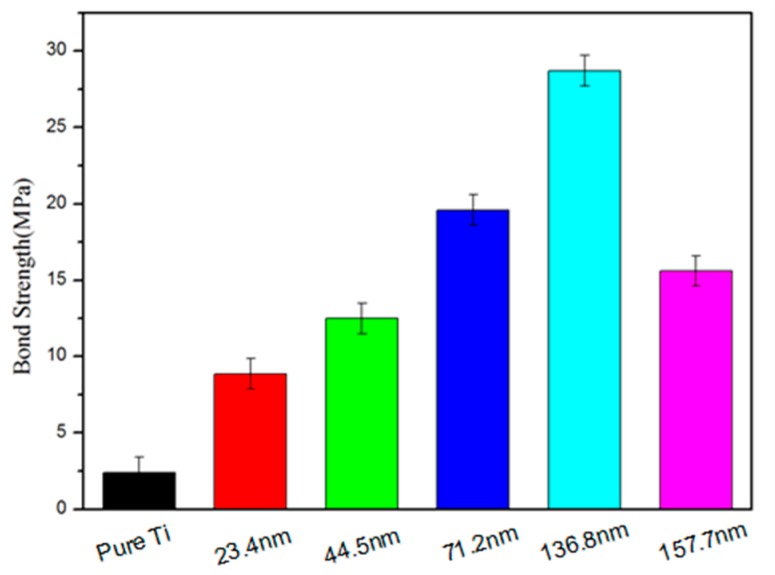
The bond strength of HA coatings on TiO_2_ nanotubes with different inner diameters.

The results of the bond strength test are shown in Figure 7. The bond strength of a layer of HA coating alone is only about 2.41 MPa. However, the bond strength of a layer of HA coating on TiO_2_ nanotubes was significantly higher. The bond strength of HA coating on TiO_2_ nanotubes with an inner diameter of 23.4 nm was 8.87 MPa; on TiO_2_ nanotubes with an inner diameter of 44.5 nm it was 12.5 MPa. The higher bond strength of 19.6 MPs for the HA on the TiO_2_ nanotubes was achieved when the inner diameter of the nanotubes reached 71.2 nm. The highest bond strength was 28.7 MPa for the HA on the nanotubes with an inner diameter of 136.8 nm. Aparicio et al. investigated micro-rough and bioactive titanium dental implants in vivo using histometry and pull-out tests and found that the pull-out strength was 3.96 MPa [26]. This result confirmed that the TiO_2_ nanotubes can act as an anchor and enhance the mechanical interlocking between the HA coating and Ti substrates. We noted that the HA coating on the TiO_2_ nanotubes with an inner diameter of 157.7 nm exhibited a lower bond strength of 15.6 MPa. This was probably due to the oversized nanotubes having a long length and thinner tube wall thickness and, therefore, breaking easily.

**Figure 8:**
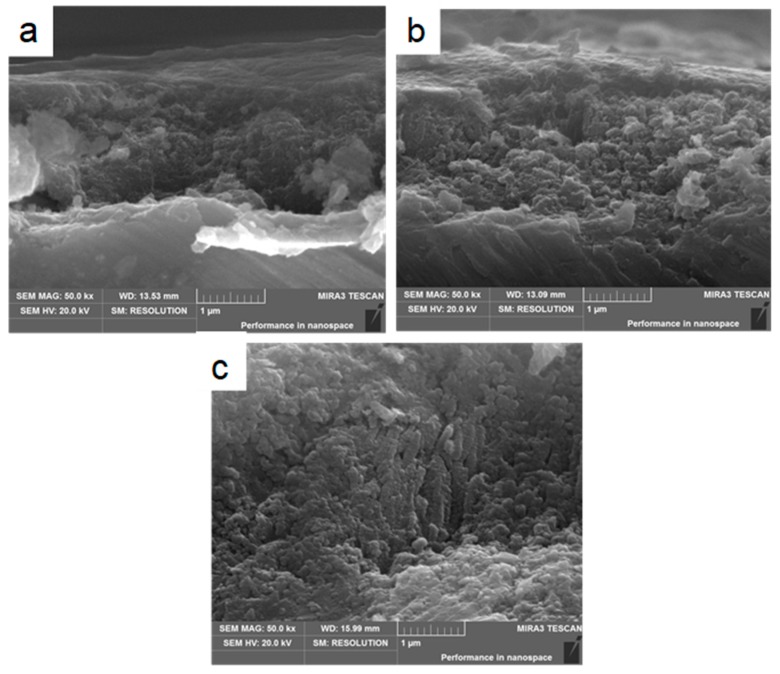
(**a**) SEM image of cross-section of HA coating on titania nanotubes anodized at 20 V for 3 h, (**b**) 20 V for 6 h, and (**c**) 20 V for 12 h.

Figure 8 shows the HA coating on TiO_2_ nanotubes with different nanotube dimensions. Figure 8a shows the cross-section of the HA coating on TiO_2_ nanotubes anodized at 20 V for 3 h. The nanotubes exhibited inner diameters of 44.5 nm, wall thicknesses of 20.0 nm and lengths of 1.25 µm. During electrochemical deposition, the particle size of the HA crystals (50 nm) was larger than the inner diameter of the TiO_2_ nanotubes (44.5 nm), and the HA crystals attached to the ends of the TiO_2_ nanotubes, rather than growing into the nanotubes. The bond strength between the HA coating and the nanotubes was weak in this deposition model. The cross-section of the HA coating on the TiO_2_ nanotubes, with inner diameters of 71.2 nm, wall thicknesses of 14.4 nm and lengths of 2.25 µm, anodized at 20 V for 6 h is illustrated in Figure 8b. The HA crystals grew into the tubes because of the larger inner diameter of the nanotubes (71.2 nm) compared to the HA particles (50 nm). The TiO_2_ nanotubes acted as an anchor [27], thus the HA nanoparticles grew into the nanotubes and formed strong mechanical interlocking between the nanotubes and the HA coating. The cross-section of the HA coating on the TiO_2_ with inner diameters of 136.8 nm, wall thicknesses of 9.8 nm and lengths of 3.94 µm anodized at 20 V for 12 h is shown in Figure 8c, the nanotube diameter is more than double the size of the HA particles, hence, the nanoparticles not only grew into the nanotubes but also grew into the gaps between the nanotubes. 

According to the literature [11,28], the growth mechanism of the anodized TiO_2_ nanotubes can be explained as follows:

(1) $$\mathrm{Ti}+2H_{2}O-4e\to {\mathrm{TiO}}_{2}+4H^{+}$$

(2) $$T{\mathrm{iO}}_{2}+6F^{-}+4H^{+}\to {\mathrm{TiF}}_{6}{}^{2-}+2H_{2}O$$

Titanium was firstly oxidized into TiO_2_ under the anodic voltage, as shown in Reaction (1). The TiO_2_ that formed on the surface of the Ti substrate reacted with F^−^ ions and dissolved when the electrolyte contained F^−^ ions, as shown in Reaction (2). Then pits formed on the Ti substrate. The formation of TiO_2_ nanotubes was the result of a dynamic circle between the electrochemical oxide and the chemical dissolution. Eventually, the pits developed into pores and grew into nanotubes.

After anodization, the HA coating was electrochemically deposited on the TiO_2_ nanotubes. Firstly, H_2_O and NH^4+^ were reduced and hydrogen gas was released. The formation of hydroxide ions led to a local increase in pH in the vicinity of the cathode (reactions are given below).

(3) $$2{\mathrm{NH}}^{4+}+2e^{-}\to H_{2}+2{\mathrm{NH}}_{3}$$

(4) $${\mathrm{NH}}_{3}+H_{2}O\to {\mathrm{NH}}_{4}{}^{+}+{\mathrm{OH}}^{-}$$

(5) $$H_{2}O+2e^{-}\to H_{2}+2{\mathrm{OH}}^{-}$$

The conditions for the deposition of brushite (CaHPO_4_·2H_2_O) were fulfilled at a pH value of ~9 through the formation of OH^−^; then HPO_4_^2−^ reacted with Ca^2+^ to produce the deposition of CaHPO_4_·2H_2_O on the surface of the TiO_2_ nanotubes, as follows:

(6) $${\mathrm{HPO}}_{4}{}^{2-}+{\mathrm{Ca}}^{2+}+2H_{2}O\to {\mathrm{CaHPO}}_{4}\cdot 2H_{2}O$$

When the coating was treated with an alkaline solution (1 M NaOH for 2 h), a hydrolysis reaction converted the brushite (CaHPO_4_·2H_2_O) into HA (Ca_10_(PO_4_)_6_(OH)_2_):

(7) $$10{\mathrm{CaHPO}}_{4}\cdot 2H_{2}O+12{\mathrm{OH}}^{-}\to {\mathrm{Ca}}_{10}{\left({\mathrm{PO}}_{4}\right)}_{6}{\left(\mathrm{OH}\right)}_{2}+4{\mathrm{PO}}_{4}{}^{3-}+30H_{2}O$$

**Figure 9:**
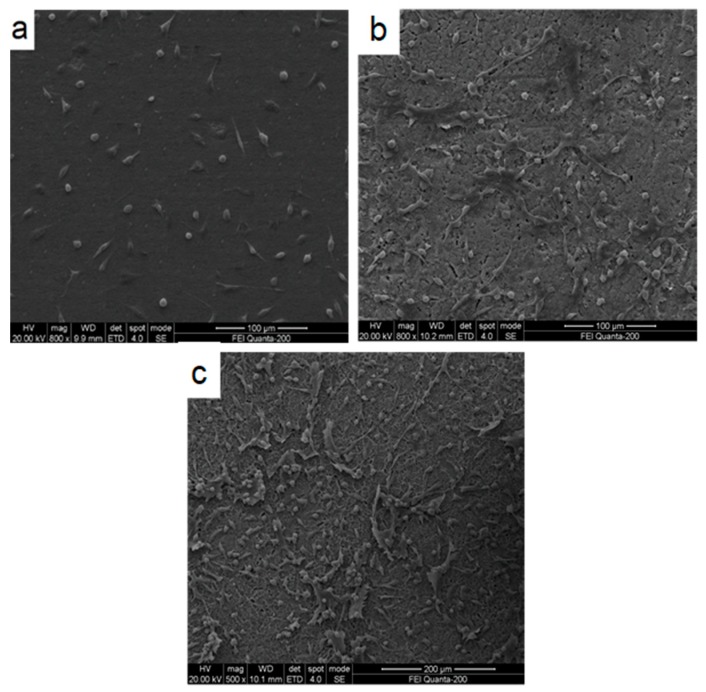
(**a**) SEM image of mesenchymal stem cells grown on uncoated Ti, (**b**) mesenchymal stem cells grown on TiO_2_ nanotubes, and (**c**) mesenchymal stem cells grown on HA coated TiO_2_ nanotubes.

The SEM images of mesenchymal stem cells cultured for 1 d on pure Ti, TiO_2_ nanotubes, and HA coating on TiO_2_ nanotubes are shown in Figure 9. We saw that the HA coating on the TiO_2_ nanotubes exhibited the highest cell numbers (Figure 9a) compared to the TiO_2_ nanotubular surface (Figure 9b) and the bare surface of pure Ti (Figure 9c), which exhibited the lowest cell numbers and no cell differentiation. Furthermore, the mesenchymal stem cells on the HA coating of the TiO_2_ nanotubes and the TiO_2_ nanotubular surface were healthier compared to those on the bare Ti surface. These cells exhibited a fusiform structure and adhered to the surface with a high density of filopodia extending out and anchored on the nanoscale characteristics of HA and TiO_2_ nanotubes. Cai et al. [29] reported that the typical morphology of healthy mesenchymal stem cells shows adhering and spreading onto coatings with some filopodial and lamellipodial extensions, indicating a high viability of the cells. Based on the morphological observations, we concluded that the nanoscale HA coating on TiO_2_ nanotubes exhibited superior biocompatibility and was more favorable for cellular activities compared to bare Ti and TiO_2_ nanotubes alone.

**Figure 10:**
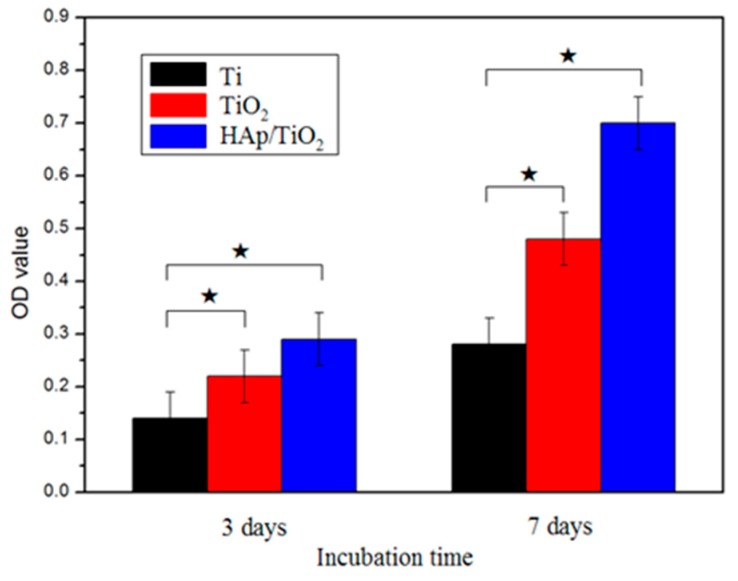
Optical density measurements showing mesenchymal stem cell proliferation on pure Ti, TiO_2_ nanotubes, and nanoscale HA coating on TiO_2_ nanotubes after culture for 3 and 7 days.

The proliferation rate of mesenchymal stem cells after culture for 3 days and 7 days are shown in Figure 10. The initial cell density seeded on bare Ti, TiO_2_ nanotubes, and the HA coating on TiO_2_ nanotubes was the same (1 × 10^4^). On Day 3, the cell number on bare Ti was significantly lower than that of the HA coated surface and the TiO_2_ nanotubes. In addition, the cell number on the HA coating on TiO_2_ nanotubes was higher than that of the TiO_2_ nanotubes. On Day 7, the HA coating exhibited greater cell growth than that of the TiO_2_ nanotubes and bare Ti. The cell viability displayed a sequence: HA on TiO_2_ nanotubes > TiO_2_ nanotubes > bare Ti. We concluded that the nanoscale HA coating on TiO_2_ nanotubes possessed excellent biocompatibility. 

## 4. Conclusions

For this paper, we fabricated a highly-ordered TiO_2_ nanotube array on the surface of a pure titanium foil using the anodization method. Then, a hydroxyapatite (HA) layer was electrochemically deposited on the vertically aligned titania (TiO_2_) nanotube array. The effect of the inner diameters on the bond strength between the nanoscale HA and the TiO_2_ nanotubes was investigated.

(1) Highly-ordered TiO_2_ nanotubes with different dimensions were successfully fabricated on titanium using the anodization method. The nanotubes were had inner diameters ranging from 44.5 to 136.8 nm, wall thicknesses ranging from 9.8 to 20.0 nm, and tube lengths ranging from 1.25 to 3.94 µm, depending on the applied potential, and the anodization time and temperature.
(2) A layer of hydroxyapatite (HA) with the average particle size of 50 nm was electrochemically deposited on to the anodized TiO_2_ nanotubes. The TiO_2_ nanotubes promoted the formation of the nanoscale HA coat by providing a high number of nucleation sites during electrochemical deposition.
(3) The dimensions of the TiO_2_ nanotubes had a significant impact on the bond strength of the HA coat on the nanotubes. The HA coat, deposited on the nanotube, with an inner diameter of 136.7 nm exhibited the highest bond strength, reaching 28.7 MPa. The TiO_2_ nanotubes with proper dimensions were favorable to the growth of HA into the nanotubes and the gaps between the nanotubes, leading to a strong mechanical interlocking effect between the HA and the nanotubes. The bond of the long oversized nanotubes with thinner tube walls could be broken easily, deteriorating the bond strength of the HA coat.
(4) A biocompatibility assessment was conducted on the Ti foil coated with the nanoscale HA on the TiO_2_ nanotube array by MTT array with mesenchymal stem cells (MSCs). The mesenchymal stem cells adhered and spread onto the nanoscale HA layer with plenty of extending filopodia, indicating the excellent biocompatibility of the HA coat, the composite coat of the nanoscale HA and the TiO_2_ nanotube array.

## References

[1] Geetha M., Singh A., Asokamani R., Gogia A. (2009). Ti based biomaterials, the ultimate choice for orthopaedic implants—A review. Prog. Mater. Sci..

[2] Rautray T.R., Narayanan R., Kwon T.Y., Kim K.H. (2010). Surface modification of titanium and titanium alloys by ion implantation. J. Biomed. Mater. Res. Part B Appl. Biomater..

[3] Hench L.L., Wilson J. (1984). Surface-active biomaterials. Science.

[4] Dorozhkin S.V. (2010). Bioceramics of calcium orthophosphates. Biomaterials.

[5] Puleo D.A., Nanci A. (1999). Understanding and controlling the bone–implant interface. Biomaterials.

[6] Zhou H., Lee J. (2011). Nanoscale hydroxyapatite particles for bone tissue engineering. Acta Biomater..

[7] Rakngarm A., Mutoh Y. (2009). Characterization and fatigue damage of plasma sprayed HAp top coat with Ti and HAp/Ti bond coat layers on commercially pure titanium substrate. J. Mech. Behav. Biomed. Mater..

[8] Abbasi S., Golestani-Fard F., Rezaie H.R., Mirhosseini S.M.M. (2012). MAO-derived hydroxyapatite/TiO_2_ nanostructured multi-layer coatings on titanium substrate. Appl. Surf. Sci..

[9] Kaviyarasu K., Mariappan A., Neyvasagam K., Ayeshamariam A., Pandi P., Rajeshwara Palanichamy R., Gopinathan C., Mola G.T., Maaza M. (2017). Photocatalytic performance and antimicrobial activities of HAp-TiO_2_ nanocomposite thin films by sol-gel method. Surf. Interface.

[10] Batory D., Gawroński J., Kaczorowski W., Niedzielska A. (2012). C–HAp composite layers deposited onto AISI 316L austenitic steel. Sur. Coat. Technol..

[11] Rath P.C., Besra L., Singh B.P., Bhattacharjee S. (2012). Titania/hydroxyapatite bi-layer coating on Ti metal by electrophoretic deposition: Characterization and corrosion studies. Ceram. Int..

[12] Mohseni E., Zalnezhad E., Bushroa A.R. (2014). Comparative investigation on the adhesion of hydroxyapatite coating on Ti–6Al–4V implant: A review paper. Int. J. Adhes..

[13] Mahdavi M., Ahmad M.B., Haron M.J., Namvar F., Nadi B., Rahman M.Z., Amin J. (2013). Synthesis, surface modification and characterisation of biocompatible magnetic iron oxide nanoparticles for biomedical applications. Molecules.

[14] Vatanpour V., Madaeni S.S., Moradian R., Zinadini S., Astinchap B. (2012). Novel antibifouling nanofiltration polyethersulfone membrane fabricated from embedding TiO_2_ coated multiwalled carbon nanotubes. Sep. Purif. Technol..

[15] Rana D., Matsuura T. (2010). Surface modifications for antifouling membranes. Chem. Rev..

[16] Durgalakshmi D., Rakkesh R.A., Balakumar S. (2015). Stacked Bioglass/TiO_2_ nanocoatings on titanium substrate for enhanced osseointegration and its electrochemical corrosion studies. Appl. Surf. Sci..

[17] Lin C.M., Yen S.K. (2006). Biomimetic growth of apatite on electrolytic TiO_2_ coatings in simulated body fluid. Mater. Sci. Eng. C.

[18] Awad N.K., Edwards S.L., Morsi Y.S. (2017). A review of TiO_2_ NTs on Ti metal: Electrochemical synthesis, functionalization and potential use as bone implants. Mater. Sci. Eng. C.

[19] Park H., Kim W.-R., Jeong H.-T., Lee J.-J., Kim H.-G., Choi W.-Y. (2011). Fabrication of dye-sensitized solar cells by transplanting highly ordered TiO_2_ nanotube arrays. Sol. Energy Mater. Sol. Cells.

[20] Minagar S., Berndt C.C., Wang J., Ivanova E., Wen C. (2012). A review of the application of anodization for the fabrication of nanotubes on metal implant surfaces. Acta Biomater..

[21] ISO 10993–5:2009 (2009). Biological Evaluation of Medical Devices—Part 5: Tests for in Vitro Cytotoxicity.

[22] Webster T.J., Ergun C., Doremus R.H., Siegel R.W., Bizios R. (2000). Specific proteins mediate enhanced osteoblast adhesion on nanophase ceramics. J. Biomed. Mater. Res..

[23] Kodama A., Bauer S., Komatsu A., Asoh H., Ono S., Schmuki P. (2009). Bioactivation of titanium surfaces using coatings of TiO_2_ nanotubes rapidly pre-loaded with synthetic hydroxyapatite. Acta Biomater..

[24] Santos A., Aw M.S., Bariana M., Kumeria T., Wang Y., Losic D. (2014). Drug-releasing implants: Current progress, challenges and perspectives. J. Mater. Chem. B.

[25] Yan Y., Zhang X., Huang Y., Ding Q., Pang X. (2014). Antibacterial and bioactivity of silver substituted hydroxyapatite/TiO_2_ nanotube composite coatings on titanium. Appl. Surf. Sci..

[26] Aparicio C., Padrós A., Gil F.-J. (2011). In vivo evaluation of micro-rough and bioactive titanium dental implants using histometry and pull-out tests. J. Mech. Behav. Biomed. Mater..

[27] Fielding G.A., Roy M., Bandyopadhyay A., Bose S. (2012). Antibacterial and biological characteristics of silver containing and strontium doped plasma sprayed hydroxyapatite coatings. Acta Biomater..

[28] Zhang D.C., Lin J.G., Wen C. (2014). Influences of recovery and recrystallization on the superelastic behavior of a β titanium alloy made by suction casting. J. Mater. Chem. B.

[29] Cai Y.L., Zhang J.J., Zhang S., Venkatraman S.S., Zeng X.T., Du H.J., Mondal D. (2010). Osteoblastic cell response on fluoridated hydroxyapatite coatings: The effect of magnesium incorporation. Biomed. Mater..

